# Persistence of immunological memory as a potential correlate of long-term, vaccine-induced protection against Ebola virus disease in humans

**DOI:** 10.3389/fimmu.2023.1215302

**Published:** 2023-09-01

**Authors:** Chelsea McLean, Karin Dijkman, Auguste Gaddah, Babajide Keshinro, Michael Katwere, Macaya Douoguih, Cynthia Robinson, Laura Solforosi, Dominika Czapska-Casey, Liesbeth Dekking, Yvonne Wollmann, Ariane Volkmann, Maria Grazia Pau, Benoit Callendret, Jerry Sadoff, Hanneke Schuitemaker, Roland Zahn, Kerstin Luhn, Jenny Hendriks, Ramon Roozendaal

**Affiliations:** ^1^Janssen Vaccines and Prevention, Leiden, Netherlands; ^2^Janssen Research and Development, Beerse, Belgium; ^3^Bavarian Nordic, Martinsried, Germany

**Keywords:** Ebola, vaccine, immunological memory, persistence, correlate, protection

## Abstract

**Introduction:**

In the absence of clinical efficacy data, vaccine protective effect can be extrapolated from animals to humans, using an immunological biomarker in humans that correlates with protection in animals, in a statistical approach called immunobridging. Such an immunobridging approach was previously used to infer the likely protective effect of the heterologous two-dose Ad26.ZEBOV, MVA-BN-Filo Ebola vaccine regimen. However, this immunobridging model does not provide information on how the persistence of the vaccine-induced immune response relates to durability of protection in humans.

**Methods and results:**

In both humans and non-human primates, vaccine-induced circulating antibody levels appear to be very stable after an initial phase of contraction and are maintained for at least 3.8 years in humans (and at least 1.3 years in non-human primates). Immunological memory was also maintained over this period, as shown by the kinetics and magnitude of the anamnestic response following re-exposure to the Ebola virus glycoprotein antigen via booster vaccination with Ad26.ZEBOV in humans. In non-human primates, immunological memory was also formed as shown by an anamnestic response after high-dose, intramuscular injection with Ebola virus, but was not sufficient for protection against Ebola virus disease at later timepoints due to a decline in circulating antibodies and the fast kinetics of disease in the non-human primates model. Booster vaccination within three days of subsequent Ebola virus challenge in non-human primates resulted in protection from Ebola virus disease, i.e. before the anamnestic response was fully developed.

**Discussion:**

Humans infected with Ebola virus may benefit from the anamnestic response to prevent disease progression, as the incubation time is longer and progression of Ebola virus disease is slower as compared to non-human primates. Therefore, the persistence of vaccine-induced immune memory could be considered as a potential correlate of long-term protection against Ebola virus disease in humans, without the need for a booster.

## Introduction

Since Ebola virus disease (EVD) was discovered in 1976, outbreaks have continued to occur with increasing frequency in sub-Saharan Africa (1). The 2014–2016 EVD epidemic in Guinea, Liberia, and Sierra Leone remains the largest outbreak to date, with over 11,000 deaths and more than 28,000 confirmed cases. The second largest outbreak occurred only 2 years later in 2018 in the Democratic Republic of Congo and Uganda, underscoring how easily EVD outbreaks can escalate to become epidemics (1).

Clinical trials have shown that the Ad26.ZEBOV, MVA-BN-Filo regimen, which is licensed for use in the European Union and six African countries, is safe and immunogenic and induces both antibody and T-cell responses in adults and children (2–15). Thus far, it has not been feasible to collect classical clinical efficacy data for Ad26.ZEBOV, MVA-BN-Filo in humans. However, the likelihood of clinical benefit was established via an immunobridging approach.

Immunobridging is a statistical analysis that translates human immunogenicity data into the likelihood of protection. This is performed by first establishing how the immune response in non-human primates (NHPs) is associated with the likelihood of protection against lethal Ebola virus (EBOV) challenge and then comparing human vaccine-induced immune responses with the NHP vaccine-induced immune response to infer the likelihood of protection in humans. The intramuscular EBOV Kikwit NHP challenge model is considered stringent, as it is 100% lethal, compared to an average human case fatality rate of 50% during Ebola outbreaks, as reported by the WHO (16). NHPs also have both a shorter incubation time (average of 5.4 days compared to 6.2–9.7 days in humans) and an extremely rapid disease progression with a shorter time to death (after symptom onset, mean survival time in NHPs is 1.4 days relative to 5.8–14.4 days to death for lethal human cases) (16, 17).

It was observed that EBOV glycoprotein (GP) binding antibody levels strongly correlated with vaccine-induced protection against EBOV in NHPs (17). Therefore, a logistic regression model was built using survival outcome as the dependent variable and the EBOV GP-binding antibody levels at 21 days post-dose 2 as the independent variable, using NHP data (n = 66) from four independent challenge studies. Survival probabilities were then estimated based on human Phase 2/3 immunogenicity data assessed at 21 days post-dose 2, using the same EBOV GP-binding antibody ELISA that was validated for both human and NHP serum testing at Q^2^ Solutions (18). To evaluate whether the vaccine regimen was likely to provide a protective benefit in humans, the lower limit of the confidence interval (CI) of the mean predicted survival probability was compared with a pre‐specified success criterion of 20%. The immunobridging analysis demonstrated a mean predicted survival probability of 53.4% with a lower limit of the pre-planned 98.7% CI of 33.8%, thereby passing the pre-defined success criterion of 20% (18) and indicating that the regimen is likely to confer a protective effect in humans.

The immunobridging model is based only on levels of circulating binding antibodies at 21 days post-dose 2 of the primary vaccine regimen. After completion of the primary vaccine regimen, a vaccine-induced immune memory response is established over time. This is evidenced by a sharp increase in EBOV GP-binding antibody levels within 7 days of re-exposure to the EBOV GP antigen via a booster dose of Ad26.ZEBOV, which indicates that a strong and rapid anamnestic response is activated upon re-exposure to the EBOV GP antigen (6, 14, 15). The disease course of EVD in the NHP model is expected to limit the contribution of an anamnestic response to protection. In contrast, an anamnestic response is expected to contribute to protection in humans because of the longer incubation time and slower disease progression of EVD. This also implies that the high-dose, intramuscular EBOV challenge in NHPs (compared to the primary route of mucosal exposure in humans) may not be a good model for the durability of protection, as protection will likely be underestimated and may no longer correlate with circulating antibody levels. Thus, immunobridging based on NHP studies can inform on the likelihood of a vaccine protective effect in humans, although there is no straightforward approach to derive the extent and the duration of the conferred benefit.

In the current manuscript, we explore whether the persistence of immunological memory could be considered as a correlate of long-term, vaccine-induced protection against EVD in humans. We analyze the persistence of the primary immune response and the persistence of immunological memory in both humans and NHPs, as demonstrated via an anamnestic response. However, alternative assessments of immunological memory, such as B-memory cell ELISpot, multi-color flow cytometry, or single-cell transcriptomics, could potentially be used in a real-life setting. We show that the onset of an anamnestic response following re-exposure to the EBOV GP antigen via booster vaccination in NHPs provides protection against EVD within 3 days. Based on the longer incubation time and slower disease progression after symptom onset in humans as compared to NHPs, the persistence of immunological memory in humans could be considered as a putative correlate of long-term protection against EVD (16, 17).

## Materials and methods

### Ethics

All clinical study protocols were conducted following the Declaration of Helsinki and International Council for Harmonisation Good Clinical Practice Guidelines (ICH-GCP) and were approved by both local and national independent ethics committees, as well as institutional review boards (IRBs) (6–11, 14, 15).

All adult participants supplied written informed consent before enrolment. For pediatric participants, parents or guardians provided written informed consent for their child to join the trial. Older children (age varied by country) also gave written assent.

### Clinical sample immunogenicity evaluations

Seven late-development studies were selected for inclusion in this analysis: EBL2001, EBL2002, Partnership for Research on Ebola VACcination (PREVAC; hereafter referred to as EBL2004), EBL2011, EBL3001 (Stage 1 was open label, while Stage 2 was randomized and active controlled), EBL3002, and EBL3003 (6–11, 14, 15). In each study, participants received an intramuscular injection of Ad26.ZEBOV on day 1 (5 × 10^10^ viral particles [vp]; dose 1) followed by an intramuscular injection of MVA-BN-Filo on day 57 (1 × 10^8^ infectious units [InfU]; dose 2).

With the exception of EBL2004, the EBOV GP-binding antibody concentration at day 21 post-dose 2 (MVA-BN-Filo) and 12 months or more post-dose 1 (Ad26.ZEBOV) was assessed. In the EBL2004 study, EBOV GP-binding antibody response was assessed at day 28 post-dose 2 and 12 months post-dose 1.

The clinical EBOV GP-binding antibody data reported here are from samples analyzed using the same validated Filovirus Animal Nonclinical Group (FANG) ELISA assay, performed at a single analytical laboratory (Q^2^ Solutions, San Juan Capistrano, CA, USA). This allows immune responses to be compared more reliably across different studies. All data are from participants who received the primary, heterologous, two-dose Ad26.ZEBOV, MVA-BN-Filo vaccine regimen with dosing on days 1 and 57. Participants in a subset of the studies additionally received a booster dose of Ad26.ZEBOV, at varying time points relative to dose 1 (Ad26.ZEBOV) of the primary regimen.

Results of the current analysis are presented by study, and results from adults and pediatric participants are presented separately. Within the studies that included pediatric participants, results are further stratified by age group. In EBL2002 and EBL3001 Stage 2 (9, 10), the EBOV GP-binding antibody response was assessed in adolescents aged 12–17 years and older children aged 4–11 years. In EBL3001 Stage 2 only, EBOV GP-binding antibody response was additionally assessed in younger children aged 1–3 years. Study EBL2004 assessed EBOV GP-binding antibody response in adolescents aged 12–17 years, older children aged 5–11 years, and younger children aged 1–4 years (11).

### Statistics

In each study, the analysis set for immunogenicity was the per-protocol set and included all vaccinated participants who had no major protocol deviations that could have influenced the immune response, had received both vaccinations within the protocol-defined window, and had at least one evaluable post-vaccination immunogenicity sample.

Antibody concentrations, in EU/mL, for the Ad26.ZEBOV, MVA-BN-Filo-vaccinated and matched control groups were summarized at each time point as geometric mean concentrations (GMCs) with corresponding 95% CIs. Responder rates were also reported for each time point post-baseline. Data from the matched control groups are not discussed in this manuscript but can be found in the original publications (6–11, 14).

For all studies, a responder was defined as a participant with an EBOV GP-binding antibody concentration >2.5-fold increased from baseline if the baseline sample was positive, or >2.5 times the lower limit of quantification (LLOQ) of 36.11 ELISA units (EU)/mL if the baseline sample was negative. For all applicable studies, outcomes for adults and pediatric participants were assessed separately.

Additional details on statistical methods, as well as baseline demographic characteristics, can be found in the original publications for each study (6–11, 14, 15).

### Non-human primate studies

All NHP studies described utilized a cynomolgus macaque (*Macaca fascicularis*) animal model. Animals were between ~3 years and 5 years of age, weighing between ~3 kg and 6 kg with an approximate 1:1 ratio between males and females. Depending on the study, the vaccination phase took place at Alpha Genesis (Yemassee, SC, USA), Bioqual (Rockville, MD, USA), or Charles River (Reno, NV, USA). For all studies, the EBOV challenge occurred at the Texas Biomedical Research Institute (TBRI, San Antonio, TX, USA). Approval of each institute’s Institutional Animal Care and Use Committee (IACUC) was obtained prior to the commencement of each study. All facilities involved were accredited by the Association for Assessment and Accreditation of Laboratory Animal Care (AAALAC), and all animal experiments performed complied with the Guide for the Care and Use of Laboratory Animals and the Animal Welfare Act regulations. While animals originated from different vendors and had different origins, this did not appear to affect vaccination or challenge outcomes.

NHPs received specialized, commercially available primate chow on a daily basis, and drinking water was available *ad libitum*. During the vaccination phase of the long-term studies, animals were socially housed. Throughout the studies, animals were provided with cage enrichment in the form of food and non-food items. In all EBOV challenge studies, humane endpoints were predefined to limit potential discomfort.

#### Study to assess immune memory activation with Ad26.ZEBOV booster vaccination

The vaccination phase of this study was performed at Alpha Genesis. Cynomolgus macaques (n = 12, Chinese origin) were obtained from the breeding colony at Alpha Genesis, except for two animals that were imported from Guangxi Grandforest Scientific Primate Company, Ltd. (Guangxi, China). Prior to the study start, animals tested negative for *Mycobacterium tuberculosis*, Simian immunodeficiency virus, Simian T-lymphotropic virus, Simian retrovirus, and herpes B virus. NHPs received an intramuscular (IM) vaccination with 5 × 10^10^ vp of Ad26.ZEBOV at study day 0, followed by an IM immunization with 1 × 10^8^ InfU of MVA-BN-Filo at study day 56 (8 weeks later). NHPs were subsequently divided into two groups of n = 6, with one group receiving no further injections and the other group receiving a booster vaccination with Ad26.ZEBOV at study day 196 (0.5 years). Both groups were observed for an additional 50 weeks to assess the activation and persistence of the memory response by booster vaccination.

#### Study to assess immune memory activation after late EBOV challenge

The group described above that did not receive the booster vaccination of Ad26.ZEBOV was transferred to TBRI for EBOV challenge 548 days (1.5 years) after the first immunization. A negative control group was included, consisting of animals originating from Alpha Genesis (n = 2, mock vaccinated with Tris-buffered saline) and animals (Vietnamese origin, n = 6, unvaccinated) obtained from Covance Research Products (Alice, TX, USA). Animals were observed for 21 days after the challenge to assess the long-term protective efficacy of the clinical regimen and activation of the memory response following the late EBOV challenge.

#### Study to assess immune memory activation after early EBOV challenge

The vaccination phase of this study was performed at Bioqual. Cynomolgus macaques (n = 4, Mauritian origin) were obtained from PrimGen (Hines, IL, USA) and acclimatized for 6 weeks before the study started. All animals tested seronegative for *M. tuberculosis*, Simian immunodeficiency virus, Simian T-lymphotropic virus, Simian retrovirus, and herpes B virus. NHPs were vaccinated IM with 5 × 10^10^ vp of Ad26.ZEBOV at study day 0, followed by an IM immunization with 1 × 10^8^ InfU of MVA-BN-Filo at study day 56 (8 weeks later). For the EBOV challenge at TBRI on study day 84 (3 months), the group that had received IM injections with empty Ad26 and MVA vectors was included as the negative control (n = 2). Animals were observed for 28 days after the challenge to evaluate the activation of the memory response after early EBOV infection.

#### Study to assess protection after booster vaccination with Ad26.ZEBOV

The vaccination phase of this study was performed at Charles River Laboratories. Cynomolgus macaques (n = 15, Mauritian origin) were obtained from Bioqual. Prior to the study’s start, animals were negative for *M. tuberculosis* and Simian retrovirus. Animals were divided into three groups of n = 5, and on study day 0, animals received either an IM vaccination with 4 × 10^10^ vp of Ad26.ZEBOV (Group 1) or 1.2 × 10^11^ vp of Ad26.Filo (Groups 2 and 3). On study day 56, all groups were vaccinated IM with 5 × 10^8^ InfU MVA-BN-Filo. After transfer to TBRI and prior to the EBOV challenge, animals received an IM booster vaccination with 4 × 10^10^ vp of Ad26.ZEBOV either 7 days (Groups 1 and 2) or 3 days (Group 3) before the challenge at study day 592 (1.62 years). The negative control group (n = 2) consisted of animals that received mock immunizations with 0.9% NaCl at study days 42 and 56, as well as 7 days prior to the challenge. After the EBOV challenge, animals were observed for 21 days to monitor the protective efficacy of an anamnestic response conferred by the booster vaccinations. One animal from both Groups 1 and 3 had to be taken out of the study due to health issues unrelated to vaccination or EBOV challenge and were therefore not included in the dataset.

#### EBOV challenge

After arrival at TBRI, animals were acclimatized to BSL-4 laboratory conditions for at least 5 days prior to EBOV challenge. For all studies, animals were exposed to a target dose of 100 plaque-forming units (PFU) (actual dose range 75.5–96 PFU) of the FANG-approved EBOV Kikwit-9510621 strain via intramuscular injection in the deltoid muscle of the right arm. For each study, animals were exposed in order of their TBRI identifier number. After the challenge, animals were observed twice daily, 7 days a week, for their health status and clinical signs of EBOV infection, with observation frequency increasing as clinical signs became apparent. Clinical manifestations were scored via an in-house scoring system, assessing general appearance, condition of skin and fur, nose/mouth/eyes/head, respiration, feces and urine, food intake, petechiae, temperature, and locomotor activity. Animals reaching a clinical score ≥15 or an otherwise moribund state were euthanized after veterinary approval. All TBRI staff were blinded to animal vaccination.

### NHP vaccines and challenge material

Ad26.ZEBOV (Janssen Vaccines and Prevention, Leiden, the Netherlands) is a recombinant, replication-incompetent, Ad26-vectored vaccine encoding the EBOV Mayinga GP. Ad26.Filo (Janssen Vaccines and Prevention, Leiden, the Netherlands) is a 1:1:1 mixture of three Ad26-vectored vaccines encoding the EBOV Mayinga variant GP, the Sudan Gulu GP, or the Marburg Angola GP. MVA-BN-Filo (Bavarian Nordic, Hellerup, Denmark) is a recombinant, modified vaccinia Ankara-vectored vaccine, non-replicating in human cells, encoding the EBOV Mayinga, Sudan Gulu, Marburg Musoke GPs, and the nucleoprotein of the Tai Forest virus. All vaccine preparations were tested for sterility and the presence of endotoxins.

EBOV strain Kikwit-9510621, supplied by TBRI, was used for all challenges. A second-cell culture passage (P2) of EBOV Kikwit was obtained from Dr. Tom Ksiazek (at the National Institute of Allergy and Infectious Diseases (NIAID) World Reference Center for Emerging Viruses and Arboviruses (WRCEVA) at the University of Texas Medical Branch (UTMB) Health Galveston National Laboratory) in 2012 and propagated at TBRI. The stock virus was passaged for a third time in Vero E6 cells to generate the challenge stock. The challenge stock was confirmed to be wild-type Ebola virus by deep sequencing and determined to be sterile and free of mycoplasma and endotoxins.

### NHP blood collection and processing

Animals were bled for serum collection at predefined time points. Blood was collected in clotting tubes, processed for isolation of serum, and subsequently aliquoted and stored at −80°C on the day of collection. Post-challenge sera were transferred to the University of Texas Medical Branch for inactivation of EBOV via a validated gamma irradiation procedure before shipment and analysis of EBOV GP binding antibodies.

### EBOV plaque assay using NHP serum

Serum viral load was determined via the FANG-optimized plaque assay for EBOV (19). In brief, frozen serum aliquots were thawed, serially diluted, and added to pre-seeded Vero E6 cells. After approximately 1 hour at 37°C, an agarose overlay medium was added to the wells and allowed to solidify. Plates were incubated for another 7 days before staining with a secondary overlay medium supplemented with neutral red to visualize the plaques. Plaques were counted 24–48 hours after staining. Serum EBOV concentration was calculated as plaque-forming units per milliliter serum (PFU/mL). Serum samples with countable plaques below the lower limit of quantification (LLOQ = 15 PFU/mL) were set at the LLOQ. Samples with 0 PFU/mL were set at 1 to enable graphing on a logarithmic scale. Data are presented in **Supplementary Figure 1**.

### Anti-EBOV GP ELISA using NHP serum

The concentration of EBOV GP binding antibodies in NHP serum was determined via the EBOV GP FANG ELISA at Battelle Biomedical Research Centre (OH, USA). The method was described previously by Rudge et al. (20). Binding antibody concentration was calculated as ELISA units per milliliter (EU/mL) serum based on a reference sample. For all data points, a median EU/mL was generated based on a minimum of two independent analyses that passed all acceptance criteria. Values below the limit of detection (LOD) were set at the LOD of each assay before log10 transformation and graphing.

## Results

### Persistence of humoral immune response following Ad26.ZEBOV, MVA-BN-Filo vaccination in adults

**Figure 1:**
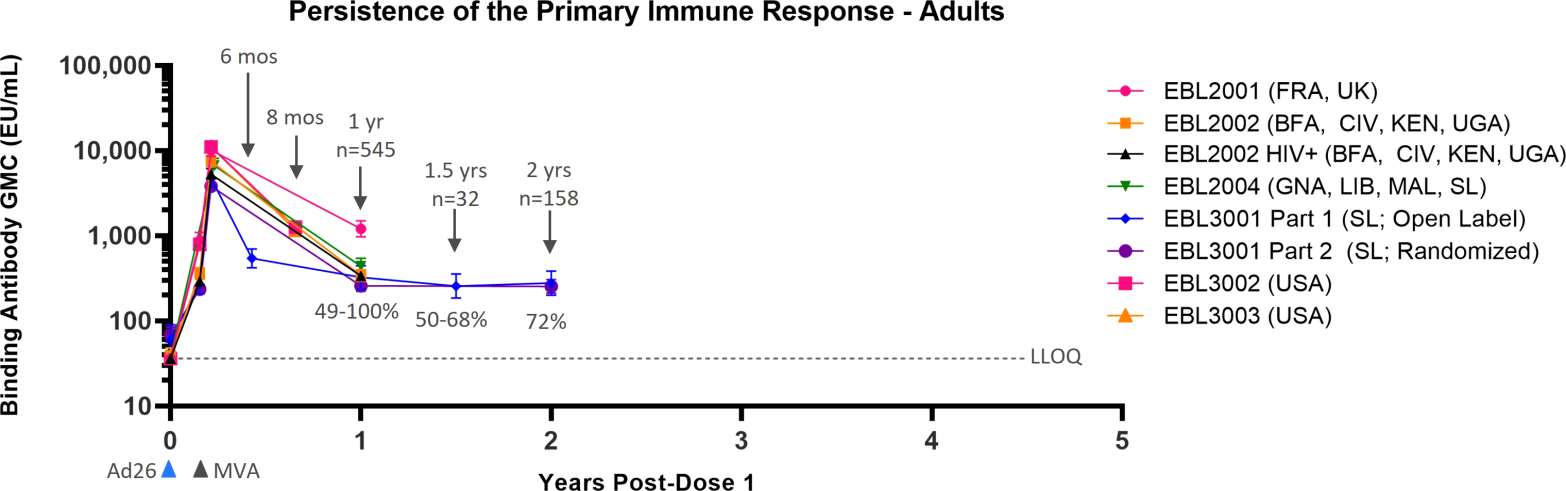
Persistence of the primary immune response in adults after vaccination with the Ad26.ZEBOV, MVA-BN-Filo vaccine regimen. EBOV GP-binding antibody GMCs in EU/mL at various time points, with accompanying 95% CIs, are depicted. Samples were analyzed following standard operating procedure at Q^2^ Solutions using the FANG ELISA, and a single reportable value for each sample at each time point was uploaded for statistical analysis. The horizontal dashed line indicates the FANG ELISA lower limit of quantification of 36.11 EU/mL. The blue arrowheads below the x-axis indicate the timing of administration of the Ad26.ZEBOV vaccine, and the black arrowheads indicate timing of administration of the MVA-BN-Filo vaccine dose. CI, confidence interval; EBOV GP, Ebola virus glycoprotein; ELISA, enzyme-linked immunosorbent assay; EU, ELISA unit; mL, milliliter; FANG, Filovirus Animal Nonclinical Group; GMC, geometric mean concentration; HIV+, human immunodeficiency virus positive; Ad26, Ad26.ZEBOV; MVA, MVA-BN-Filo; Mos, months; Yr, year; Yrs, years; FRA, France; UK, United Kingdom; BFA, Burkina Faso; CIV, Côte d’Ivoire; KEN, Kenya; UGA, Uganda; GNA, Guinea; LIB, Liberia; MAL, Mali; SL, Sierra Leone; USA, United States of America. N is the total number of participants, from all studies, with data at the indicated time point. Percentages indicate the range of percent responders observed across studies at the indicated time point.

Data from six Phase 2/3 clinical studies, all analyzed with the same validated FANG ELISA assay performed in the same laboratory, are available to support the persistence of immune response to vaccination in adults, with the majority of studies including a time point of 1 year post-dose 1 (**Table 1**). Circulating EBOV GP-binding antibody GMCs decline between 21 days post-dose 2 and 6 months post-dose 2 (8 months post-dose 1), at which point a plateau is reached, with some variation in binding antibody GMCs by geographic location (**Figure 1**). At 1 year post-dose 1, circulating binding antibody GMCs persisted from 259 EU/mL in the randomized, active controlled Stage 2 of EBL3001 in Sierra Leone to 1,205 EU/mL in the UK and France in EBL2001. Responder rates at 1 year ranged from 49% in EBL3001 to 100% in several studies. Study EBL3001 also included a 2-year post-dose 1 time point, where circulating binding antibody GMCs were 279 EU/mL and 255 EU/mL in Stage 1 (open-label) and Stage 2 (randomized, active controlled), respectively. This is comparable to the GMCs observed at 1 year and 1.5 years post-dose 1 within this study, indicating that further decay is slow once the plateau phase is reached.

**Table 1:** Persistence of Ebola virus glycoprotein circulating binding antibody response in adults.

	Study (age strata)	Persistence time point analyzed	N	GMC (EU/mL)	% Persisting response
				(95% CI)	
**Phase 2** **(Q^2^ Solutions)**	**EBL2001 (FRA, UK)** ***Healthy adults***	1 year post-dose 1 (Day 365)	50	**1,205** (971; 1,497)	100%
	**EBL2002 (BFA, CIV, KEN, UGA)** ***Healthy adults***	1 year post-dose 1 (Day 365)	133	**342** (291; 401)	78%
	**EBL2002 (BFA, CIV, KEN, UGA)** ***HIV-infected adults***	1 year post-dose 1 (Day 365)	59	**338** (253; 450)	88%
	**EBL2004 (GNA, LIB, MAL, SL)** ***Healthy adults***	1 year post-dose 1 (Day 365)	254	**437** (352; 542)	80%
**Phase 3** **(Q^2^ Solutions)**	**EBL3001, Stage 1 (SL)** ***Healthy adults***	1 year post-dose 1 (Day 360)	31	**325** (238; 445)	77%
	**EBL3001, Stage 2 (SL)** ***Healthy adults***	1 year post-dose 1 (Day 360)	168	**259** (223; 301)	49%
	**EBL3001, Stage 1 (SL)** ***Healthy adults***	2 years post-dose 1 (Day 720)	31	**279** (201; 386)	68%
	**EBL3001, Stage 2 (SL)** ***Healthy adults***	2 years post-dose 1 (Day 720)	158	**255** (212; 306)	50%
	**EBL3002 (USA)** ***Healthy adults***	8 months post-dose 1 (Day 237)	131	**1,263** (1,100; 1,450)	99%
	**EBL3003 (USA)** ***Healthy adults***	8 months post-dose 1 (Day 237)	244	**1,151** (~950; ~1400)	98%

GMC, geometric mean concentration; EU/mL, enzyme-linked immunosorbent assay units per milliliter; CI, confidence interval; FRA, France; UK, United Kingdom; BFA, Burkina Faso; CIV, Côte d’Ivoire; KEN, Kenya; UGA, Uganda; GNA, Guinea; LIB, Liberia; MAL, Mali; SL, Sierra Leone; USA, United States of America.

### Persistence of humoral immune response following Ad26.ZEBOV, MVA-BN-Filo vaccination in children and adolescents

**Figure 2:**
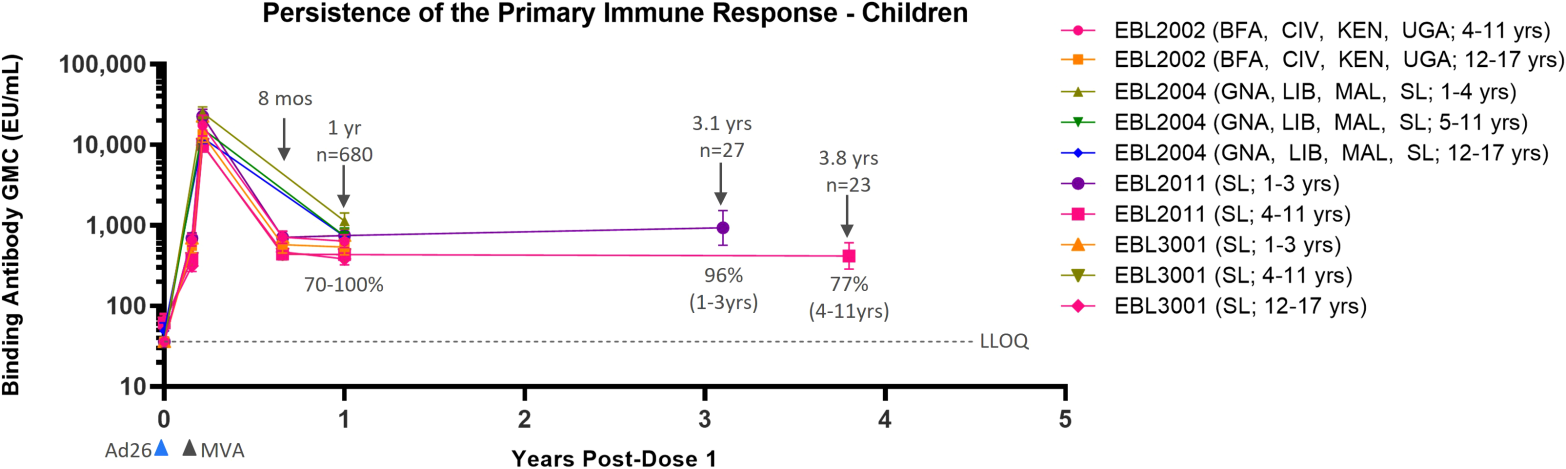
Persistence of the primary immune response in children and adolescents after vaccination with the Ad26.ZEBOV, MVA-BN-Filo vaccine regimen. EBOV GP-binding antibody GMCs in EU/mL at various time points, with accompanying 95% CIs, are depicted. Samples were analyzed following standard operating procedure at Q^2^ Solutions using the FANG ELISA, and a single reportable value for each sample at each time point was uploaded for statistical analysis. The horizontal dashed line indicates the FANG ELISA lower limit of quantification of 36.11 EU/mL. The blue arrowheads below the x-axis indicate the timing of administration of the Ad26.ZEBOV vaccine, and the black arrowheads indicate timing of administration of the MVA-BN-Filo vaccine dose. CI, confidence interval; EBOV GP, Ebola virus glycoprotein; ELISA, enzyme-linked immunosorbent assay; EU, ELISA unit; mL, milliliter; FANG, Filovirus Animal Nonclinical Group; GMC, geometric mean concentration; Ad26, Ad26.ZEBOV; MVA, MVA-BN-Filo; Mos, months; Yr, year; Yrs, years; BFA, Burkina Faso; CIV, Côte d’Ivoire; KEN, Kenya; UGA, Uganda; GNA, Guinea; LIB, Liberia; MAL, Mali; SL, Sierra Leone. N is the total number of participants, from all studies, with data at the indicated time point. Percentages indicate the range of percent responders observed across studies at the indicated time point.

Data from four Phase 2/3 clinical studies in pediatric participants show similar results as compared to adults (**Table 2**). Immune response as measured by EBOV GP-binding antibody GMCs decline between 21 days post-dose 2 and 6 months post-dose 2 (8 months post-dose 1). After this point, circulating binding antibody levels reach a plateau (**Figure 2**). At 1 year post-dose 1, circulating binding antibody GMCs ranged from 386 EU/mL in adolescents in Sierra Leone in EBL3001 to 1139 EU/mL in children 1–4 years old in Guinea, Liberia, Mali, and Sierra Leone in EBL2004. Responder rates at 1 year post-dose 1 ranged from 70% to 100%, and responses tend to be higher at 1 year post-dose 1 in younger individuals, both compared to adults and between the age group stratifications for pediatric participants. This plateau of circulating binding antibody levels is maintained up to at least 3.1 years in children aged 1–3 years at the time of dose 1 vaccination (934 EU/mL [568–1,534]; 96%) and at least up to 3.8 years in children aged 4–11 years at the time of dose 1 vaccination (418 EU/mL [287–608]; 77%). These results indicate that circulating binding antibodies persist, with minimal additional decline, for at least 3.8 years post-dose 1.

**Table 2:** Persistence of Ebola virus glycoprotein circulating binding antibody response in children and adolescents.

	Study (age strata)	Persistence time point analyzed	N	GMC (EU/mL) (95% CI)	% Persisting response
**Phase 2** **(Q^2^ Solutions)**	**EBL2002 (BFA, CIV, KEN, UGA)** ***4–11 years***	1 year post-dose 1 (Day 365)	53	**638** (529; 767)	98%
	**EBL2002 (BFA, CIV, KEN, UGA)** ***12–17 years***	1 year post-dose 1 (Day 365)	54	**541** (433; 678)	90%
	**EBL2004 (GNA, LIB, MAL, SL)** ***1–4 years***	1 year post-dose 1 (Day 365)	105	**1139** (905; 1432)	100%
	**EBL2004 (GNA, LIB, MAL, SL)** ***5–11 years***	1 year post-dose 1 (Day 365)	109	**739** (585; 933)	94%
	**EBL2004 (GNA, LIB, MAL, SL)** ***12–17 years***	1 year post-dose 1 (Day 365)	127	**731** (589; 907)	77%
	**EBL2011 (SL)** ***1–3 years***	3.2 years post-dose 1 (Day 1168)	27	**934** (568; 1534)	96%
	**EBL2011 (SL)** ***4–11 years***	3.2 years post-dose 1 (Day 1168)	23	**418** (287; 608)	77%
**Phase 3** **(Q^2^ Solutions)**	**EBL3001 (SL)** ***1–3 years***	1 year post-dose 1 (Day 360)	120	**750** (629; 894)	96%
	**EBL3001 (SL)** ***4–11 years***	1 year post-dose 1 (Day 360)	123	**436** (375; 506)	71%
	**EBL3001 (SL)** ***12–17 years***	1 year post-dose 1 (Day 360)	132	**386** **(326; 457)**	70%

GMC, geometric mean concentration; EU/mL, enzyme-linked immunosorbent assay units per milliliter; CI, confidence interval; BFA, Burkina Faso; CIV, Côte d’Ivoire; KEN, Kenya; UGA, Uganda; GNA, Guinea; LIB, Liberia; MAL, Mali; SL, Sierra Leone; USA, United States of America.

### Persistence of immune memory following Ad26.ZEBOV, MVA-BN-Filo primary regimen vaccination and Ad26.ZEBOV booster vaccination in adult and pediatric participants

**Figure 3:**
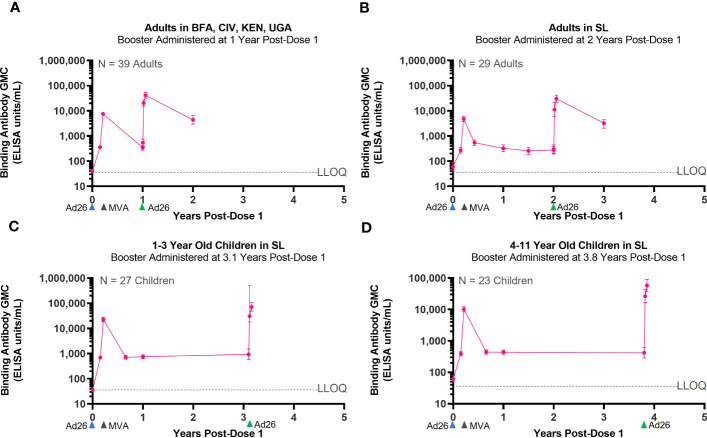
Persistence of the primary immune response after vaccination with the Ad26.ZEBOV, MVA-BN-Filo vaccine regimen and activation of an immune memory response after administration of an Ad26.ZEBOV booster dose. EBOV GP-binding antibody GMCs in EU/mL at various time points, with accompanying 95% CIs, are depicted. Ad26.ZEBOV booster dose administered between 1 year and 3.8 years post-dose 1 in adults **(A, B)** and children **(C, D)**. Samples were analyzed following standard operating procedure at Q^2^ Solutions using the FANG ELISA, and a single reportable value for each sample at each time point was uploaded for statistical analysis. The horizontal dashed lines indicate the FANG ELISA lower limit of quantification of 36.11 EU/mL. The blue arrowheads below the x-axis indicate the timing of administration of the Ad26.ZEBOV vaccine doses, the black arrowheads indicate timing of administration of the MVA-BN-Filo vaccine dose, and the green arrowheads indicate timing of administration of the Ad26.ZEBOV booster dose. CI, confidence interval; EBOV GP, Ebola virus glycoprotein; ELISA, enzyme-linked immunosorbent assay; EU, ELISA unit; mL, milliliter; FANG, Filovirus Animal Nonclinical Group; GMC, geometric mean concentration; Ad26, Ad26.ZEBOV; MVA, MVA-BN-Filo; Yrs, years; BFA, Burkina Faso; CIV, Côte d’Ivoire; KEN, Kenya; UGA, Uganda; GNA, Guinea; LIB, Liberia; MAL, Mali; SL, Sierra Leone. N is the number of participants with data at pre-booster baseline.

Three Phase 2/3 clinical studies included the administration of an Ad26.ZEBOV booster dose at various time points after completion of the primary Ad26.ZEBOV, MVA-BN-Filo vaccine regimen. These studies were conducted in adults (EBL2002, EBL3001) and pediatric participants (EBL2011). In each study, the Ad26.ZEBOV booster was administered at a different time points, ranging from 1 year to 3.8 years, after dose 1 of the primary regimen (**Table 3**). Regardless of when the Ad26.ZEBOV booster dose was administered, a strong anamnestic immune memory response was observed, as indicated by a sharp increase in EBOV GP-binding antibody GMCs within 7 days (**Figure 3**). The fold increase in EBOV GP-binding antibody GMCs from pre-booster to 7 days post-booster ranged from approximately 33-fold when the booster was administered at 3.1 years post-dose 1 in 1–3-year-old pediatric participants in EBL2011 to 63-fold in 4–11-year-old pediatric participants in EBL2011 when the booster was administered at 3.8 years post-dose 1 (**Table 4**).

**Table 3:** Overview of clinical studies which administered a booster dose of Ad26.ZEBOV.

Study phase	Study number	Location	Population	Method	Booster dose administration
**Phase 2**	**EBL2002**	Burkina Faso, Côte D’Ivoire, Kenya Uganda	• Healthy adults • HIV-infected adults • 4–11 years • 12–17 years	Randomized, placebo-controlled, observer-blind	• 1 year
	**EBL2011**	Sierra Leone	• 1–3 years • 4–11 years	Open label	• 3.1 years • 3.8 years
**Phase 3**	**EBL3001**	Sierra Leone	• Healthy adults	Staged study with an open-label, uncontrolled Stage 1 followed by a randomized, controlled, double-blind Stage 2	• 2 years

HIV, human immunodeficiency virus.

**Table 4:** Activation of immune memory response in humans after administration of an Ad26.ZEBOV booster dose.

		21 days post-dose 2	Pre-booster	7 days post-booster		21 days post-booster		
**Column**		**A**	**B**	**C**	**C:B**	**D**	**D:B**	**D:A**
**Study** **(age strata)**	**Timing of booster administration relative to dose 1 of the primary regimen**	GMC* (EU/mL)	**GMC**	**GMC**	**Fold increase**	**GMC**	**Fold increase**	**Fold increase:** **21 days post-booster** vs. **21 days post-dose 2**
EBL2002	1 year	**7,518**	**366**	**20,416**	**55.8**	**41,643**	**113.8**	**5.5**
EBL3001 Stage 1	2 years	**4,784**	**274**	**11,166**	**40.8**	**30,411**	**111**	**6.4**
EBL3001/EBL2011 (1–3 years)	3.1 years (1–3 years)	**22,568**	**934**	**30,463**	**32.6**	**71,143**	**76.2**	**3.2**
EBL3001/EBL2011 (4–11 years)	3.8 years (4–11 years)	**10,212**	**418**	**26,478**	**63.3**	**57,564**	**137.7**	**5.6**

GMC, geometric mean concentration; EU/mL, enzyme-linked immunosorbent assay per milliliter.

*GMC of all participants at 21 days post-dose 2 in the indicated study or parent study of the indicated study as applicable.

GMCs continued to rise when assessed at 21 days post-booster relative to day 7, followed by a decline when assessed at 1 year post-booster. At 21-days post-booster, the fold changes from pre-booster increased to between 76-fold when the booster was administered at 3.1 years post-dose 1 in 1–3-year-old pediatric participants in EBL2011 and to 138-fold from pre-booster when the booster was administered at 3.8 years post-dose 1 in 4–11-year-old pediatric participants in EBL2011 (**Table 4**). When the anamnestic response at 21 days post-booster was compared to the immune response observed at 21 days post-dose 2 of the primary regimen, a 3.2-fold increase was observed if the booster was administered at 3.1 years post-dose 1 in pediatric participants, and this was increased to 5.6-fold if the booster was administered at 3.8 years post-dose 1 in pediatric participants (**Table 4**).

In terms of persistence of the anamnestic immune response post-booster in adults, circulating binding antibodies were still detectable at 1 year post-booster in studies EBL2002 (booster administered at 1 year post-dose 1) and EBL3001 (booster administered at 2 years post-dose 1). Responder rates in these two studies were 97% at 1 year post-booster in study EBL2002 and 100% at 1 year post-booster in study EBL3001. Additionally, circulating binding antibody GMCs at 1 year post-booster were approximately 12 times higher than the GMCs observed at 1 year post-dose 1 (**Table 5**).

**Table 5:** Persistence of immune memory response in humans after administration of an Ad26.ZEBOV booster dose.

		1 year post-dose 1	Pre-booster	1 year post-booster		
**Column**		**A**	**B**	**C**	**C:B**	**C:A**
**Study** **(age strata)**	**Timing of booster administration relative to dose 1 of the primary regimen**	GMC*	**GMC** **(EU/mL)**	**GMC** **(EU/mL)**	**Fold increase**	**Fold increase: 1 year post-booster** vs. **1 year post-dose 1**
EBL2002	1 year	**342**	**366**	**4,383**	**12**	**12.8**
EBL3001 Stage 1	2 years	**279**	**274**	**3,237**	**11.8**	**11.6**
EBL3001/EBL2011 (1–3 years)	3.1 years	**750**	**934**	**NA**	**NA**	**NA**
EBL3001/EBL2011 (4–11 years)	3.8 years	**436**	**418**	**NA**	**NA**	**NA**

GMC, geometric mean concentration; EU/mL, enzyme-linked immunosorbent assay per milliliter.

*GMC of all participants at 1 year post-dose 1 in the indicated study or parent study of the indicated study (as applicable).

### Persistence of humoral immune responses and immune memory following Ad26.ZEBOV, MVA-BN-Filo primary regimen in NHPs

**Figure 4:**
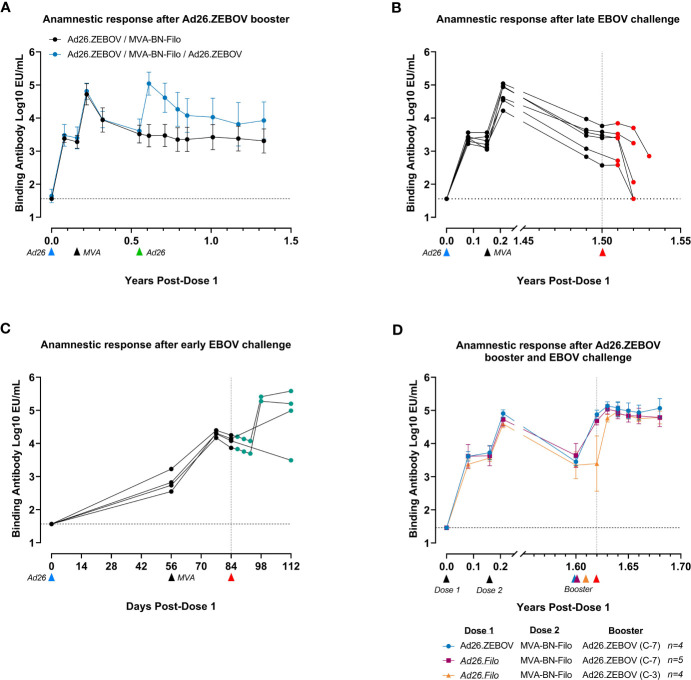
Anti-EBOV GP responses in serum of NHPs after vaccination and EBOV challenge as a measure of vaccine immunogenicity and the anamnestic response after challenge. EBOV GP-binding antibody levels at various time points in log10 EU/mL at various time points. **(A)** Comparison of the antibody response over time after vaccination with the clinical regimen with or without Ad26.ZEBOV boost at 0.5 years (day 196). Data are shown as group mean with standard deviation, n = 6/group. **(B)** Serum antibody concentration after vaccination followed by EBOV challenge 1.6 years (548 days) after the first dose. Individual NHP response profiles are shown. **(C)** Serum antibody concentrations after vaccination with Ad26.ZEBOV and MVA-BN-Filo, followed by short-term EBOV challenge 84 days after the first dose. Individual NHP response profiles are shown. The x-axis for this panel is reported in days, rather than years, due to the short time course of the experiment. **(D)** Serum antibody concentrations after vaccination with either Ad26.ZEBOV-MVA-BN-Filo or Ad26.Filo-MVA-BN-Filo, followed by Ad26.ZEBOV booster either 7 days or 3 days prior to EBOV challenge, 1.6 years (592 days) post-first dose. Data are displayed as group mean with standard deviation; group sizes as indicated in the figure legend. C-7, booster administration 7 days prior to EBOV challenge; C-3, booster administration 3 days prior to challenge. In **(A–C)**, the blue arrows below the x-axis indicate the timing of administration of the Ad26.ZEBOV vaccine doses, and the black arrows indicate timing of administration of the MVA-BN-Filo vaccine dose. In all panels, red arrows indicate intramuscular challenge with EBOV. Data points shaded green represent animals surviving EBOV challenge, while data points shaded red represent animals succumbing to the challenge before the study end. Dashed horizontal lines indicate lower limit of detection for each data set. For data in **(C)** the LOD for post-challenge data was set at the LOD of the pre-challenge data (1.46 vs. 1.56 log10 EU/mL). A continuous x-axis is used for panels **(A, C)** and an interrupted x-axis is used for **(B, D)** EU/mL, ELISA units per milliliter; GP, glycoprotein; NHPs, non-human primates; LOD, limit of detection.

The kinetics of humoral immune responses in NHPs were similar to those observed in humans. The immune response, as measured by circulating EBOV GP-binding antibody levels, declined between 21 days post-dose 2 and 6 months post-dose 2 (8 months post-dose 1) (**Figure 4A**), after which a stable plateau phase was reached that persisted for at least 17 months (~1.4 years). A booster dose of Ad26.ZEBOV (5 × 10^10^ vp) administered 4 months after the two-dose primary regimen elicited an anamnestic immune memory response, indicated by an approximately 40-fold increase in EBOV GP-binding antibody levels by day 7 post-booster. Antibody levels again declined from day 21 post-booster onward and reached a plateau level approximately fourfold higher than after the two-dose primary regimen. Thus, it is clear that immunological memory was also maintained in NHPs, which could rapidly be re-activated by exposure to the EBOV GP vaccine antigen.

### Exposure to EBOV activates an anamnestic response, but disease progress in NHPs is too rapid for protection

Based on the kinetics of the anamnestic response and the speed of disease progression in NHPs, it appeared unlikely that the anamnestic response would outcompete disease progression in the NHP model (100 pfu IM infection leads to lethality in approximately 7 days). Indeed, when a cohort of six NHPs was challenged IM with EBOV approximately 1.5 years after the first immunization, all animals succumbed to infection (**Supplementary Figure 1A**). When EBOV GP-binding antibodies were analyzed post-challenge, an anamnestic response was not observed (**Figure 4B**), indicating that the persistent level of circulating antibodies at this time point was not sufficient, and the NHPs succumbed before an effective memory response could be mounted. Indeed, starting at day 6 post-challenge, antibody titers declined in all animals, reaching undetectable levels in two animals before they succumbed to EVD. Circulating antibodies were depleted by excess GP production due to viral replication and did not confer sufficient protection in the absence of an anamnestic response.

Contrastingly, when NHPs vaccinated with Ad26.ZEBOV, MVA-BN-Filo were infected with EBOV early after vaccination (4 weeks after dose 2) when levels of persistent circulating binding antibodies are higher, a substantial increase in EBOV GP-binding antibody levels was observed in three out of four NHPs from day 14 post-challenge (**Figure 4C**). This proves that EBOV exposure can elicit an anamnestic response in NHPs when disease progression is delayed. While some animals displayed clinical signs, none became viremic, and all survived until the study end (**Supplementary Figure 1B**), suggesting that circulating binding antibodies were able to delay disease progression long enough for the anamnestic response to contribute to protection.

### Pre-activation of the anamnestic response elicits rapid protection in NHPs

To investigate whether an anamnestic response to the EBOV GP antigen could contribute to protection in the NHP model, a cohort of immunized NHPs received a booster vaccination 7 days or 3 days prior to the challenge to simulate immune memory activation. A cohort of NHPs were immunized with either Ad26.ZEBOV as dose 1 or Ad26.Filo as dose 1. Ad26.Filo is a 1:1:1 mixture of three Ad26-vectored vaccines encoding the EBOV GP, the Sudan virus GP, or the Marburg virus GP. Thus, Ad26.Filo contains the same EBOV GP antigen as Ad26.ZEBOV. All animals, regardless of whether Ad26.ZEBOV or Ad26.Filo was administered as dose 1, received MVA-BN-Filo as dose 2 in a 56-day interval. This cohort received a booster (Ad26.ZEBOV) approximately 1.6 years after dose 2 and was challenged with EBOV either 7 days or 3 days later. Irrespective of the preceding regimen, all NHPs that were re-exposed to the EBOV GP antigen by way of an Ad26.ZEBOV booster 7 days or even 3 days prior to EBOV infection survived the challenge, with minimal morbidity and absence of viremia (**Supplementary Figure 1C**). In agreement with previous data (**Figure 4A**), a booster immunization 7 days prior to the challenge resulted in a fully developed, protective anamnestic response at the time of challenge (**Figure 4D**). A booster immunization 3 days prior to the challenge did not result in a detectable increase in EBOV GP-binding antibody levels by the time of challenge, but by day 3 post-challenge, a protective anamnestic response had developed. Thus, in NHPs, re-exposure to the EBOV GP antigen via a booster immunization provides protection within 3 days.

### Permission to reuse and copyright

No copyrighted material from other sources (including the web) is included in this manuscript.

## Discussion

Vaccination with Ad26.ZEBOV, MVA-BN-Filo administered in a 56-day interval in humans induces strong EBOV GP-binding antibody responses that persist for at least 3.8 years post-dose 1. This agrees with modeling data, which suggest that antibody concentrations could persist, with minimal decline, up to 5 years after initial vaccination (21). Immunological memory is also maintained up to at least 3.8 years post-dose 1 and can be activated within 7 days by a booster immunization with Ad26.ZEBOV to levels greater than the highest levels observed after dose 2 of the primary vaccine regimen. After booster immunization, antibody levels sharply increase before declining and appear to be stable at 1 year post-booster at levels 12-fold higher than the plateau that persisted after the primary two-dose regimen. The kinetics of Ad26.ZEBOV, MVA-BN-Filo-induced EBOV GP-binding antibody responses are very similar when comparing humans with NHPs. After an initial peak, antibody levels in NHPs decline and reach a plateau phase approximately 6 months after dose 1 that persists for at least 1.3 years post-dose 1. An immunological memory response can be rapidly activated by a booster immunization with Ad26.ZEBOV, with GP-binding antibody levels exceeding levels reached at 21 days post-dose 2 of the primary regimen already observed within 7 days after the booster. Infection with EBOV also increased GP-binding antibody levels in animals infected shortly after vaccination, indicating that infection can activate an anamnestic response when at least partial protection is provided by the primary response. However, due to the rapid progression of EVD in the NHP model, there was no sufficient time for this anamnestic response to confer protection after a late EBOV challenge. An anamnestic response triggered via a booster immunization provided an onset of protection within 3 days, which is prior to a strong increase in circulating EBOV GP-binding antibodies. We will now first discuss the role of circulating EBOV GP-binding antibodies in protection against EVD before turning to the potential contribution of the anamnestic response to protection.

Early studies implicated CD8^+^ T-cell responses in EVD protection mediated by an adenovirus type 5 (Ad5)-based vaccine (22), and innate immune responses were implicated in early protection by rVSV (23). However, a remarkably consistent picture emerges, across a wide range of vaccine platforms, that circulating EBOV GP-binding antibodies correlate with protection against EVD. This was indeed observed for vaccines based on vesicular stomatitis virus (VSV) (24), Ad5 (22), chimpanzee adenovirus type 3 (ChAd3) (25), virus-like particles (VLPs) (26), Ad26 and MVA (17), parainfluenza virus (PIV), and Newcastle disease virus (NDV) vectored vaccines (27). At least in some cases, antibody functionality appeared to be more closely associated with protection when compared to EBOV GP-binding antibody level per se (27). Circulating EBOV GP-binding antibody levels are therefore potentially a surrogate of an underlying mechanism of protection, such as Fc-receptor binding and neutrophil phagocytosis, which were associated with persistent protection after rVSVΔG-ZEBOV (28). In our studies, the level of circulating EBOV GP-binding antibodies after vaccination with Ad26.ZEBOV, MVA-BN-Filo was strongly correlated with the level of EBOV-neutralizing antibodies and had a similar discriminatory capacity for predicting challenge outcomes in NHPs, while cellular responses were independently correlated with protection (17). In addition, Ad26.ZEBOV, MVA-BN-Filo vaccination also induced antibody effector functions in humans, as shown by antibody-dependent NK-cell activation (29). As yet, it is unclear to what extent different antibody-mediated effector mechanisms are involved in protection against EVD by Ad26.ZEBOV, MVA-BN-Filo, though the long-term presence of Fc-gamma receptor binding antibodies in Sudan virus (SUDV) survivors suggests that these types of antibodies could be important for protection against infection (30). Similarly, it was observed that EBOV survivors have high levels of antibody-dependent cellular cytotoxicity and antibody-dependent cellular phagocytosis as compared to rVSV vaccinated individuals (31). In conclusion, circulating EBOV GP-binding antibodies likely contribute to protection elicited by virtually all GP-based vaccines, though they are also correlated with other mechanisms of protection.

The fact that the EBOV GP-binding antibody level is not necessarily a mechanistic correlate for protection imposes restrictions on when it can be used to infer protection. For instance, it is not a given that the correlation between circulating EBOV GP-binding antibody levels and survival is quantitatively similar at all time points after vaccination, even within a single vaccine platform. This is clearly illustrated by an elegant experiment that explored the onset of protection against MARV, using a ChAd3-based vaccine (32). Though there was a strong correlation between MARV GP-binding antibody levels 4 weeks after vaccination and protection 5 weeks after vaccination, the vaccine provided an onset of protection within 1 week in the absence of detectable MARV GP-binding antibody levels. In our own studies, protection was observed within 3 days after a booster vaccination in NHPs (**Figure 4D**), while circulating EBOV GP-binding antibodies were below levels that are associated with protection early after vaccination (17). This suggests that the initiation of the anamnestic response, rather than the level of EBOV GP-binding antibodies, could be considered as a potential correlate of protection in NHPs at later time points after vaccination.

In terms of the potential contribution of the anamnestic response to protection at later time points after vaccination, the triggering of the anamnestic response in NHPs needed to be supported by a booster vaccination with Ad26.ZEBOV given just before the challenge. However, the slower progression of EVD in humans could permit enough time for an anamnestic response to be triggered by the GP produced upon EBOV infection. Thus, the vaccine protective effect could evolve over time, being strongly correlated with circulating EBOV GP-binding antibody levels early after vaccination, while the durability of the protective effect would rely on persistent immunological memory (18). A similar situation exists for smallpox, where the vaccine take, as identified by a skin reaction, is considered the best predictor of vaccine efficacy, also highlighting a role for a protective memory response (33). In addition, in the case of hepatitis B, a full course of vaccination confers complete protection against acute clinical disease and chronic hepatitis B infection for long periods of time based on persisting memory responses, even after circulating antibody responses have become undetectable (34). Data from clinical studies with Ad26.ZEBOV, MVA-BN-Filo show that circulating EBOV GP-binding antibody concentrations in humans plateau approximately 6 months post-dose 2, and this plateau is maintained for at least 3.8 years. Although the tentative protective threshold for EBOV GP-binding antibodies of 200 EU/mL was identified for a different vaccine platform, which may have a different correlate of protection, it is interesting to note that the levels of circulating EBOV GP-binding antibodies persisting at 3.8 years after dose 1 of the Ad26.ZEBOV, MVA-BN-Filo vaccine regimen (418 EU/mL) were higher than this threshold. Importantly, administration of a booster dose of Ad26.ZEBOV resulted in a strong and rapid immune memory response, within 7 days of booster administration. This immune memory response was persistent over time and could be re-activated even when the booster dose was administered 3.8 years after dose 1 of the primary vaccine regimen.

The only filovirus vaccine for which a putative correlate of human protection has been identified is the rVSVΔG-ZEBOV vaccine (35). Both circulating EBOV GP-binding and neutralizing antibodies were correlated with protection, with EBOV GP-binding antibodies providing a better differentiation between protected and non-protected individuals. An EBOV GP-binding antibody level of 200 EU/mL was tentatively identified as a protective threshold in humans. This remarkably low level of EBOV GP-binding antibodies would not likely give sterilizing immunity on its own, providing further support for the notion that in humans, a vaccine anamnestic response contributes to protection against EVD, similar to the smallpox vaccine mentioned above. If this is indeed the case for EBOV infection, it may eventually be possible to establish persistent memory as a correlate of protection, irrespective of the vaccine platform, as most vaccine-mediated protection is based on the Ebola GP antigen. Thus, vaccines that have independently established protective efficacy in the NHP model could be evaluated for persistent immunological memory in humans while acknowledging potentially divergent correlates of protection (36). EBOV infection also triggered an anamnestic response in NHPs, albeit with apparently slower kinetics. The slower kinetics after EBOV infection versus an Ad26.ZEBOV booster may be due to a combination of immune modulation by EBOV (37), the time needed for viral replication to reach EBOV GP levels capable of triggering the anamnestic response, and soluble GP produced by EBOV reducing the amount of measurable circulating GP-binding antibodies. Taking into consideration the incubation time and slower disease progression in humans versus NHPs, it is likely that a protective anamnestic response could be mounted upon natural exposure to EVD, even several years after primary vaccination. Therefore, the ability to activate such an immune memory response could be considered as a potential independent correlate of long-term protection against EVD in humans.

## Data availability statement

The raw data supporting the conclusions of this article will be made available by the authors, without undue reservation. Janssen has an agreement with the Yale Open Data Access (YODA) Project to serve as the independent review panel for the evaluation of requests for clinical study reports and participant-level data from investigators and physicians for scientific research that will advance medical knowledge and public health. Data will be made available following publication and approval by YODA of any formal requests with a defined analysis plan. For more information on this process or to make a request, please visit the Yoda Project site at http://yoda.yale.edu. The data sharing policy of Janssen Pharmaceutical Companies of Johnson & Johnson is available at https://www.janssen.com/clinical-trials/transparency.

## Ethics statement

The studies involving humans were approved by The French national Ethics Committee (CPP Ile de France III; 3287), the French Medicine Agency (150646A-61), the UK Medicines and Healthcare Products Regulatory Agency (MHRA), and the UK National Research Ethics Service (South Central, Oxford; A 15/SC/0211) (EBL2001); the Burkina Faso Central Ethics Committee, the Comite National D’Ethique de La Recherche (Guinea), the Kenyatta National Hospital/University of Nairobi Ethical Review Committee (Kenya), the Uganda Virus Research Institute Research Ethics Committee, and the Makerere University School of Public Health Research and Ethics Committee (Uganda) (EBL2002); Comité d’Evaluation Ethique de l’Inserm, the London School of Hygiene & Tropical Medicine Ethics Committee the Comité National d’Ethique pour la Recherche en Santé (Guinea), National Health Science Research Ethics Committee (Liberia), the University of Mali Faculty Med Pharmacy & Dentistry (Mali); and the Sierra Leone Ethics and Scientific Review Committee (Sierra Leone) (EBL2004); The Sierra Leone Ethics and Scientific Review Committee, the Pharmacy Board of Sierra Leone, and the London School of Hygiene & Tropical Medicine Ethics Committee (EBL2011); the Sierra Leone Ethics and Scientific Review Committee, the Pharmacy Board of Sierra Leone, and the London School of Hygiene and Tropical Medicine Ethics Committee (EBL3001); MaGil Institutional Review Board (EBL3002); MaGil Institutional Review Board (EBL3003). For pediatric participants, parents or guardians provided written informed consent for their child to join the trial. Older children (age varied by country) also gave written assent. The studies were conducted in accordance with the local legislation and institutional requirements. Written informed consent for participation in this study was provided by the participants’ legal guardians/next of kin. The animal studies were approved by the Institutional Animal Care and Use Committee (IACUC) of Alphagenesis, Bioqual, Charles River, or the Texas Biomedical Research Institute, depending on where the study was performed. The studies were conducted in accordance with the local legislation and institutional requirements. Written informed consent was not obtained from the owners for the participation of their animals in this study because the animals were purchased from commercial suppliers by Janssen. Therefore, Janssen was both the animal owner and study executor.

## Author contributions

CM, KD, and RR wrote the manuscript and were involved in the design of experiments, as well as in data acquisition, analysis, and interpretation. AG, BK, MK, MD, CR, RZ, KL, and JH were involved in the design of the experiments, as well as in data acquisition, analysis, and interpretation. All authors contributed to the article and approved the submitted version.
