# Supplementary material for: Persistence of immunological memory as a potential correlate of long-term, vaccine-induced protection against Ebola virus disease in humans
Source: Front Immunol. 2023 Sep 1;14:1215302. doi: 10.3389/fimmu.2023.1215302 (PMC10505757; doi:10.3389/fimmu.2023.1215302)
Supplement: Supplementary file 1 [file DataSheet_1.docx]

Supplementary Material

**Persistence of immunological memory as a potential correlate of long-term, vaccine-induced protection against Ebola virus disease in humans**

**Chelsea McLean, Karin Dijkman, Auguste Gaddah, Babajide Keshinro, Michael Katwere, Macaya Douoguih1, Cynthia Robinson, Laura Solforosi, Dominika Czapska-Casey, Liesbeth Dekking, Yvonne Wollmann, Ariane Volkmann, Maria Grazia Pau, Benoit Callendret, Jerry Sadoff, Hanneke Schuitemaker, Roland Zahn, Kerstin Luhn, Jenny Hendriks, and Ramon Roozendaal****

**** Correspondence:** Corresponding Author: roozendaal@zonmw.nl

# Supplementary Data

This manuscript does not include Supplementary Data.

# Supplementary Figures and Tables

## Supplementary Figures


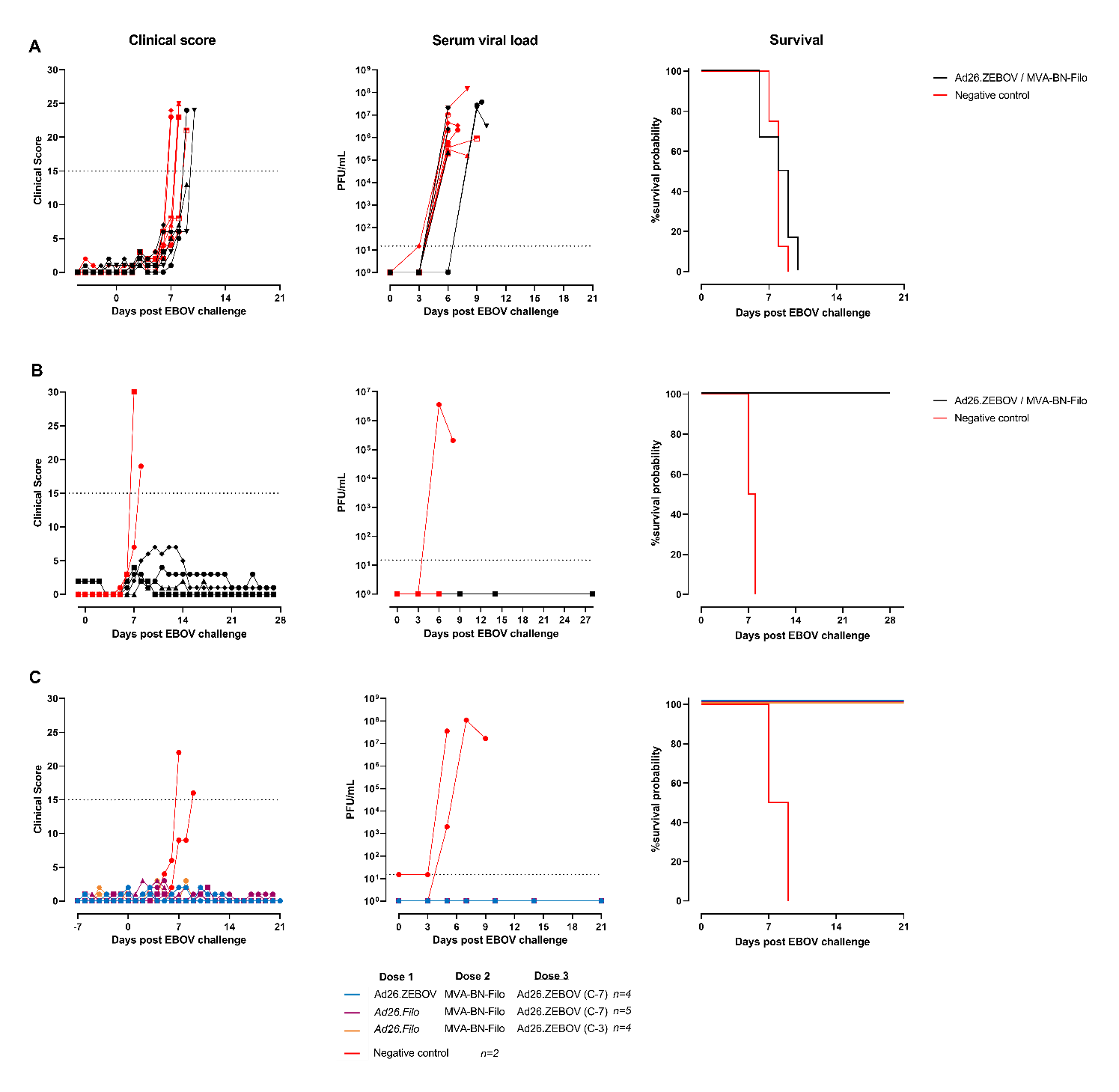


**Supplementary Figure 1. Clinical scores, serum viral load, and survival of animals after vaccination and EBOV challenge.** Clinical scores (left panels), serum viral load (middle panels), and survival probability (right panels) of vaccinated animals after exposure to a target dose of 100 PFU EBOV. A) Clinical scores, serum viral load, and survival after EBOV challenge 548 days post first dose. Ad26.ZEBOV group: n = 6, negative controls: n= 8. B) Clinical scores, serum viral load, and survival after EBOV challenge 84 days post first dose. Ad26.ZEBOV group: n = 4, negative controls: n= 6. C) Clinical scores, serum viral load, and survival after EBOV challenge 3 or 7 days post Ad26.ZEBOV boost, 592 post first dose. Group sizes are indicated in the figure. Dashed horizontal line left panels indicate the clinical score threshold for euthanasia. NHP not surviving to study end not reaching this threshold were found dead in cage. Dashed horizontal line in middle panels indicate the lower limit of quantification for the serum plaque assay (LLOQ = 15). Samples with counts below this value were set at 15, samples with no countable plaques were set at 1 to enable graphing on a logarithmic scale. PFU/mL = plaque forming units per milliliter.
